# Comparing the Information and Support Needs of Different Population Groups in Preparation for 2015 Government Approval for HIV Self-testing in France

**DOI:** 10.1371/journal.pone.0152567

**Published:** 2016-03-31

**Authors:** Tim Greacen, Delphine Kersaudy-Rahib, Jean-Marie Le Gall, Nathalie Lydié, Jade Ghosn, Karen Champenois

**Affiliations:** 1 Laboratoire de recherche, EPS Maison Blanche, Paris, France; 2 INPES, Saint-Denis, France; 3 AIDES, Pantin, France; 4 AP-HP, UF de Thérapeutique en Immuno-Infectiologie, Hôpital Hôtel-Dieu, Paris, France; 5 Université Paris Descartes, EA7327, Faculté de Médecine site Necker, Paris, France; 6 Inserm U1018, CESP-4: Epidémiologie du VIH et des IST, Le Kremlin Bicêtre, France; The University of New South Wales, AUSTRALIA

## Abstract

**Context:**

HIV self-tests are currently being introduced in France with the aim of promoting screening both for the general population and for high-risk populations.

**Objective:**

The current study aimed to identify and compare the information and support needs of the different target population groups.

**Methods:**

The Delphi process was used to synthesize expert opinions for each population group. Experts were chosen for their experience and expertise in the area of HIV and HIV screening for each population. Each group developed recommendations for a specific population: six high HIV prevalence populations (men who have sex with men; transgender people; substance users; migrants from sub-Saharan Africa; French West Indies; French Guiana) and two low prevalence populations (the general population; people under 25). Each group included expertise from four areas: research, screening and care, policy-making, and community groups.

**Results:**

A final total of 263 recommendations were grouped into eight main themes: *Communicating at both national and community levels about self-test arrival* (24% of all recommendations); *Providing information adapted to the different community groups’ needs* (23%); *Providing counselling on self-test use and access to care* (15%); *Making self-tests available to all in terms of accessibility and cost* (13%); *Preparing community healthcare and screening systems for the arrival of the self-test* (11%); *Approving only high quality self-tests* (6%); *Defending self-test users’ legal rights* (5%); *Evaluating self-test use* (3%). Although a large number of recommendations were common to several groups of experts, the study highlighted a certain number of recommendations specific to each different population group, particularly with regard to information content and access both to information and to the self-tests themselves.

**Conclusion:**

Results from the current study should make a significant contribution to policy decisions concerning catering for the specific access, information and support needs of different potential HIV self-test user groups in France.

## Introduction

Screening plays a major role in current HIV prevention strategies [1–5]. However, in France, a large number of people at risk of acquiring HIV are not being tested, or are being tested too late [6–8], with serious consequences for the people involved [7] and a significantly increased risk of onward transmission to others [1]. Indeed, despite the fact that over five million tests are performed in France every year, it is estimated that some 30,000 people, i.e. 19% of people infected with HIV, are unaware of their HIV status [9]. Amongst these, one third would be men who have sex with men (MSM), one third heterosexual migrants from sub-Saharan Africa and the remaining third, heterosexuals born in France. Thus, facilitating access to repeated screening opportunities for the more vulnerable groups with regard to HIV remains a major public health priority. Given this epidemiological context, the French Ministry of Health, based on reports by the National AIDS Council [10] and the National Ethics Committee [11], recently ruled in favor of making HIV self-tests available to the general public. The first such test went on sale on September 15, 2015.

Some highly at risk populations are clearly interested in accessing self-tests. In an online survey performed in French on HIV self-tests, 87% of the 5,908 non HIV positive MSM previously unaware of the existence of these tests expressed interest in accessing them if they were authorized [12,13]. These men were more likely to be younger, to live in smaller towns and to live with their parents or their wives and children. They also were more likely to engage in unsafe sex with casual male partners, to live their sex lives with men in absolute secrecy and yet not to have been tested for HIV recently. When asked why they were interested in accessing self-tests, respondents gave the same three main reasons already identified in the USA for this same population group: convenience, rapidity and anonymity [14]. Men not interested in accessing self-tests gave the following reasons for their lack of interest: satisfaction with current screening methods, doubts about reliability, not wanting to be alone when discovering results and fear of incorrect use [12].

Interest for self-tests is not just limited to at-risk population groups such as MSM. The general population is also interested [15–17]. In France, around 70% of respondents in a 2010 survey declared that self-tests would facilitate testing, with the younger respondents being more likely to prefer self-testing [18]. A review of the international literature by Krause et al. (2013) concluded that self-testing is highly acceptable among all populations studied, whether in resource-limited contexts (Kenya, Malawi) or in higher income countries (USA, Spain, Singapore) [19]. Participants underlined the importance of anonymity, but at the same time expressed worries about accessing counseling and care [19]. A systematic review on evidence from supervised and unsupervised strategies found, for both strategies, high levels of acceptability (74%-96%), preference for this type of test (61%-91%), and interest in doing the test with sexual partners (80%-97%) [20]. However, for coherent public health policy on making HIV self-tests publicly available, the authors of the latter study highlighted the lack of research exploring people’s motivations to use the self-test, their fears, needs, perceptions, preferences and priorities. Finally, a third more recent literature review on attitudes and acceptability of HIV self-testing for key populations concluded that most studies addressing this question have taken place in high-income countries with men who have sex with men], underlining the need for research concerning other key populations [21].

The present study aimed to address these issues using a qualitative survey with HIV screening experts working with vulnerable or less vulnerable populations with regard to HIV, and with the principle objective of identifying the information and support needs of the different groups of potential self-test users in France.

## Methods

From February to May 2014, experts working in eight parallel groups participated in a three-round Delphi process conducted on the Internet. The Delphi process collects and synthesizes expert opinion on a given issue in the area of their expertise. Participants are chosen for their expertise, and are ensured anonymity with respect to the recommendations they might make. Each expert then gives a score to all the recommendations made by the members of their group. In light of the replies of the other participants, they are then asked to revise their initial answers with the aim of bringing the group towards a consensus, or presenting clear sets of opposing arguments should points of view be discordant [22]. Three rounds are generally viewed as being sufficient for achieving a high level of agreement [23].

The experts were chosen by the study’s scientific committee for their experience and expertise in the area of HIV and HIV screening for each population. Each group developed recommendations for a specific population, including six high HIV prevalence populations: men who have sex with men (MSM), migrants from sub-Saharan Africa, substance users (including both injection and non-injection substance use), transgender people, French West Indies, French Guiana; and two low prevalence populations: the general population, people aged under 25. The choice of including a specific group on young people, in spite of their current low prevalence rates, was linked to the results of a 2010 survey [18] which revealed that today’s younger generation in France seems to be less aware of HIV risks than their elders. The procedure for choosing experts was as follows. Firstly, an extensive list of potential participants was compiled for each population group. All members of the scientific committee then scored the experts according to their perceived expertise with regard to HIV testing for the population group in question. For each population group, the representatives with the highest mean scores were invited to participate, but at the same time including at least one expert from each of the four key expert categories: researchers publishing in the area of HIV and screening; clinicians working in HIV medical services or in screening centers; policy-makers in regional or national health authorities; board members or employees of community organizations addressing the sexual health concerns of the population group in question. No expert participated in more than one group. The aim was to include a total of ten to twelve experts for each group, thus constituting a pooling of judgments, as much as possible from each of the four expert categories, as well as taking into account the information processing capability of the research team, as recommended by Hsu and Sandford [24]. The entire Delphi process was conducted by email, thus allowing a broad geographical representation: apart from the two expert groups specifically addressing the two overseas French departments with high HIV prevalence, each group included experts from the Greater Paris Area (Ile-de-France) as well as from the other provinces.

In the first round, experts were asked to propose ten or so statements that, in their opinion, constituted “good practices for responding to the information and support needs of HIV self-test users”. For each factor, experts were asked to explain briefly why they considered this to be important. The final list of factors for each expert group was then analyzed by two researchers using the following method: (a) factors that were identical or that used different words to describe the same phenomenon were grouped into one factor; (b) factors that covered more than one issue were divided into distinct entities. The researchers took pains to respect each expert’s nuances in describing his or her different recommendations using, as much as possible, the terms and expressions chosen by the experts themselves to formulate final versions of each recommendation. After the within-group analysis for each of the eight groups of experts, the same process was used to identify factors that were common to more than one expert group and to harmonize the terminology used across different groups.

In the second round, the complete list of factors identified by all the experts within each group were sent back to all the members of that group. Experts were then asked to score each factor on a scale from 1 (not at all relevant) to 9 (highly relevant) with regard to the degree of importance they attributed to that factor for informing and supporting self-test users when the self-test would be officially approved for sale and would come onto the market in France. The mean score for each factor was then calculated as the group score for that factor for the group in question.

Finally, in the third round, experts were invited to reconsider their scores, if they wished, in the light of the mean group score for each factor.

For the purposes of the present study, recommendations with scores **≥**7 out of a possible maximum of 9 were considered to be relevant. Recommendations are presented by decreasing mean score. Standard deviations are used to describe the level of agreement between experts of the same group.

The Paris Descartes University Health Research Ethics Board (CERES) discussed this project and conveyed that no ethical committee review was required for this study, the methodology used presenting no problems of any ethical nature. Participants were deemed to have consented by returning their recommendations.

## Results

Ten to twelve experts were identified and contacted for each target population. Although the participation of the experts from mainland France proceeded as planned, the response and acceptance rates of experts from the French West Indies and Guiana proved to be considerably lower, despite the repeated entreaties of the research team. Overall, from February to May 2014, a total of 72 experts completed the Delphi process, including only 6 for the French West Indies and 5 for French Guiana. The general characteristics of the experts for each expert group are presented in the Table 1.

**Table 1:** Characteristics of the experts in each population group.

Group	Number of experts	Researchers	Clinicians involved in HIV testing and/or care*	Policy-makers	Community Organizations
MSM	10	3	3	0	4
Migrants from sub-Saharan Africa	10	1	5^a^	0	4
Substance users	11	2^b^	2^a^	2	5
Transgender people	9	2	2	1	4
French West Indies	6	2	2	1	1
French Guiana	5	1^c^	1	2	1
General population	10	4	2	2	2^d^
Young people	11	2	3	4	2

* Most clinicians are qualified sexual health care providers.

^a^ One clinician also belongs to the community organization category.

^b^ One researcher is also a policy-maker.

^c^ One researcher also belongs to the community organization category.

^d^ One member of one of the community organizations is also a researcher.

Using the qualitative methods as described above, a final total of 263 recommendations were identified and aggregated into eight themes (Table 2). The two major themes concerning communication and information about the self-test and how to use it accounted for 47% of the total number of recommendations.

**Table 2:** Number of recommendations for each major theme.

	n	%
Communicating at national, community and population-specific levels concerning the self-test	62	24
Providing users with reliable, user-friendly and population-specific information on using the self-test	60	23
Providing quality support to users purchasing and using the test and accessing care in the case of a positive result	40	15
Making self-tests available to different population groups in terms of accessibility and cost	35	13
Preparing community healthcare and existing screening support and information systems before the self-test comes onto the market	28	11
Approving for sale only high quality self-tests	17	6
Defending self-test users’ legal rights	13	5
Evaluating self-test use	8	3
**Total number of recommendations**	**263**	**100**

Table 3 shows the total number of recommendations and the number of recommendations with a mean score **≥**7 for each group of experts. The MSM expert group stood out from other groups with regard to their high total number (90) of recommendations, compared to 60 and 57 respectively for the same number of experts for Substance Users and the General Population, or to 48 for the nine experts in the Transgender group. This was a first indication of the considerable interest of the self-test for MSM experts compared to the experts for other population groups. With regard to high-scoring recommendations (mean score **≥**7), the General Population expert group stood out with only 35% of recommendations with mean scores **≥**7, compared to the migrants from sub-Saharan Africa or Transgender groups with 79% and 88%, respectively.

**Table 3:** Number of recommendations for each expert group.

Expert Group	Number of experts	Total number of recommendations	Recommendations with mean score ≥7, n (%)
Men who have sex with men	10	90	53 (59%)
Migrants from sub-Saharan Africa	10	66	52 (79%)
Substance Users	11	60	38 (63%)
Transgender people	9	48	42 (88%)
French West Indies	6	55	45 (82%)
French Guiana	5	43	31 (72%)
Young people	11	77	47 (61%)
General Population	10	57	20 (35%)

### Recommendations shared by several expert groups

More than one in three (35%) recommendations were common to several groups of experts: 34 were common to four or more groups, 20 others to three groups and 38 to two groups. Three recommendations were common to all eight groups:

- *The instructions on how to use the self-test and how to interpret test results need to be clear and comprehensible for all* (mean score: m = 8.6).
- *The instructions should indicate how to interpret a negative result and understand the implications of testing during the seroconversion window period* (m = 8.2).
- *Create a telephone hotline providing support on how to use the test, accessible 24/7* (m = 7.8).

Among recommendations common to three or more groups, 76% (n = 41) were scored as relevant (mean score ≥7). These recommendations tended to address general practical questions (Table A in S1 File). Almost one out of two of these high-scoring recommendations common to three or more groups concerned the theme of: *Providing users with reliable*, *user-friendly and population-specific information on using the self-test* (19 recommendations). The three other main themes were: *Making self-tests available to different population groups in terms of accessibility and cost* (6), *Communicating at national*, *community and population-specific levels concerning the self-test* (6) and *Providing quality support to users purchasing and using the test and accessing care in the case of a positive result* (5). The highest scoring of these recommendations—*The instructions must indicate what to do in case of a positive result* (m = 8.9/9)—was common to seven of the eight expert groups.

### Priority recommendations for the eight population groups

A final list of recommendations was thus established for each of the eight groups of experts. Within each list, some recommendations were common to other groups, whereas others were specific to that one particular expert group (Tables B-I in S1 File). The following section presents a summary of the key issues for each population group, underlining recommendations that were specific to each group or for which the average score given by one particular expert group was markedly different from the score given to the same recommendation by other expert groups.

#### Men who have sex with men (MSM)

As pointed out above, the MSM expert group made the highest number of recommendations. As for the other expert groups, information and support were priority issues, particularly with regard to community support for MSM with positive test results. More specifically, these experts underlined the importance, *for population groups such as MSM with high prevalence rates and multiple risk-taking*, *of doing the test regularly*, *of repeating the test and placing less emphasis on waiting until the end of the seroconversion window after taking a risk*. Information for MSM should take into account their higher risk epidemiological context: *The instructions need to emphasize that a negative test result does not mean that your partner is negative*, *even if you had unprotected sex together; inform users about early HIV acute infection symptoms and the greater risk of transmitting the HIV virus during this phase*. Anonymity being a key question for certain MSM, the MSM experts recommended *using the campaign promoting self-tests for the general public to get messages through to hidden vulnerable population groups for whom anonymity might be a crucial issue*, such as MSM who do not frequent the gay scene or who live their MSM lives in secrecy. Almost one in five (18%) MSM recommendations addressed issues around accessing self-tests, compared to 13% for all groups, underlining the importance of diversity of access to HIV self-tests in terms of location, websites and cost. Finally, MSM experts insisted upon official marketing approval of only *the most reliable tests (in terms of sensitivity and specificity)*, *with marketing approval reviewed regularly in order to guarantee self-test quality*.

#### Migrants from sub-Saharan Africa

Three recommendations from the expert group for migrants from sub-Saharan Africa scored 9/9, giving a good indication of their major concerns: (1) *a single-use test*, reflecting their concern for questions of safety: the experts explained their recommendation by the fact that migrant populations, with limited resources, might try to use the same test a second time or on several different people; (2) *the instructions should underline the importance of knowing if you are HIV positive as early as possible*; (3) *results that are easy to understand*. Other high scoring recommendations focused on having *a test that is easy to use*, with instructions that are *clear and comprehensible for all users*, *including those who do not have a high educational level*.

The role of community organizations was considered to be essential, not only for communicating about the self-test, but also for monitoring self-test use in these migrant populations. At the same time, it was considered to be important *not to create a specific self-test access route for migrants*, *but rather create specific support strategies for migrants in vulnerable situations*. This was the only group that promoted the idea that *people selling or distributing self-tests need to do so in a positive way*. Finally, *the instructions need to make it clear that*, *in France*, *HIV care is financed entirely by the government*, *no matter what the administrative status of the person might be*. Issues around users' rights and free access to screening and care with no out-of-pocket payment were major concerns: 12% of the recommendations for migrants from sub-Saharan Africa belonged to the theme *Defending self-test users’ legal rights* compared to an overall 5% for all expert groups.

#### Substance Users (SU, including both injection and non-injection use)

For the substance user expert group, amongst the highest priorities was to *work together with substance use community organizations to develop the tools needed for making substance users aware of the possibility of doing self-tests and for setting up ways of accessing self-tests and appropriate support*.

General themes of particular importance for this expert group included *information adapted to substance users’ needs* (38% of recommendations vs. 23% for all groups) and *access to HIV self-tests* (20% vs. 13% for all groups). Experts recommended *publicity for self-tests on injection kit packaging or other harm-reduction tools*, with particular care being paid to making self-tests accessible for substance users whose experiences have led them to avoid other screening possibilities. As well as *providing self-tests free-of-charge in substance use care services*, self-tests should be made available *in all places that are in contact with substance users*. It was also suggested that substance use services should propose sending self-tests to users in the post, as certain centers already do with safer injection material. Finally, with regard to confirming self-test results, it was deemed important to *identify screening centers that know how to look after injecting substance users who would be mistrustful of a blood sample being taken by someone else*.

#### Transgender people

The transgender expert group made relatively few recommendations (48) but scored 88% of them as relevant. Almost one in three of their recommendations (31%) concerned the general theme of *Communicating at national*, *community and population-specific levels concerning the self-test*, compared to 24% for all groups.

For the transgender experts, a health and screening system that respects gender diversity was paramount: *respecting peoples’ genders; respecting the diversity of transgender people*; *fighting against conventional stereotyping*; *not forgetting female-to-male transgender people*. Furthermore, *online support staff need to be trained on the specific problems of the different transgender communities*, with access to self-tests taking into account cultural diversity and the specific problems of transgender sex workers. Although experts agreed on *demedicalizing communication addressing transgender people*, it was at the same time considered essential to promote *the right to hormonal therapy and to surgery*, and *remove administrative barriers to allow transgender people to take care of themselves better and facilitate HIV screening*. Finally, self-test *instructions must list likely interactions with substances or medication being used, mouth and teeth problems, hormonal treatment or hormone cycles and, conversely, reassure users if there are unlikely to be any problematic interactions. Access to care must not interrupt hormonal therapy*.

#### French West Indies (FWI)

Although the six experts in this group expressed relatively few recommendations compared to other expert groups, 82% of these recommendations were considered to be relevant. More than one in three recommendations (36%) were about *information on HIV self-tests* (compared to 23% for all groups). Similarly to the expert group for migrants from sub-Saharan Africa, the West Indian experts underlined the importance of *the instructions indicating the storage conditions for the self-test* and making it clear that *the self-test is a single-use test*. Anonymity was an issue: experts insisted upon users being able to *access the self-test without having to go through a health professional and creating distribution circuits that protect people’s confidentiality*, *for example using dispensers*. They also recommended *wide-scale access*, *outside healthcare centers*, *including vouchers to allow people to obtain self-tests for free in drugstores*.

#### French Guiana

Recommendations from the French Guiana expert group were often related to the specificity of the Guianese context: diversity of ethnic, linguistic and cultural composition, high proportion of immigrants (>30%), variety of economically disadvantaged communities, low population density, and tropical climate. Experts insisted on being able to access *self-tests that can be stored at room temperature* in Guianese contexts, i.e. above 30° centigrade and in high humidity. Self-test instructions and the telephone help-line must be available in the principle spoken languages, with *information that is accessible and understandable for everyone*, *including minors*, *people with low education levels*, *or who are illiterate*. It is important to avoid too much medical terminology, and to *work with cultural mediators on the terms and symbols to be used* when promoting self-tests. They also felt it to be important in Guiana *to underline the advantages of self-testing*: *practical*, *rapid and discreet*.

#### Young people (<25)

Experts for young people placed particular importance on the theme of *providing users with reliable, user-friendly and population-specific information on using the self-test* (36% of recommendations vs. 23% for all groups): *information about self-testing needs to be accessible and understandable for everyone, including minors, people with little education or who are illiterate; the instructions need to be sufficiently attractive to be read even by those who think they already know how to use the self-test*. Specifically for minors, the experts recommended that the self-test instructions should remind them about their *right to confidentiality* with regard both to self-testing and to accessing health care. Furthermore, these experts underlined the importance of *official government texts making it clear that minors have the right to access self-tests*.

High levels of disagreement were observed in this group concerning access to self-tests and the possibility for young people to obtain them free-of-charge. For example, the recommendation *The self-tests should be easy to access*, *at low cost or free-of-charge for people under 25 who refuse other forms of screening* reached a mean score of 7.4/9, but with a standard deviation of 2.5, indicating considerable disagreement between the experts involved. Similarly, *self-tests should be available free-of-charge in school infirmaries* scored 7.1/9, but with a standard deviation of 2.3. On the other hand, experts agreed for *proposing supervised self-testing; however this should be an option*: *supervision should not be a precondition for accessing the test* (m = 8.0; SD = 1.2).

In contrast to the MSM expert group, who recommended self-tests using blood for greater test reliability, this group recommended self-tests using crevicular samples, considering this to be more easily acceptable for young people.

#### General Population

As indicated above, the General Population expert group made by far the lowest number of recommendations with mean score ≥7. The only recommendations specific to this expert group were to insist upon the instructions informing users about *the importance of privacy*, *and the need to think carefully before disclosing the test result to anyone* and the proposition that the self-test should be made *available to community organizations via a central purchasing mechanism*. As for the MSM experts, they recommended approval of *only the most reliable tests (in terms of sensitivity and specificity)*, with tests being assessed regularly in order to guarantee quality self-test development. The other high scoring recommendations were all common to several groups, particularly with regard to the content of the instructions and how to access information: *indicating what to do if you get a positive test result*, providing information that is *clear and comprehensible for all users including those who do not have a high educational level*, making the instructions *sufficiently concise to be easily readable in one sitting*, *explaining how to access 24/7 free-of-charge telephone support on how to use the test*. They also recommended *a moderate and accessible price for all or even free-of-charge for population groups with greater HIV risk*. However, disagreement appeared among General Population experts concerning *providing access to self-tests elsewhere than in drugstores* (m = 6.2, SD = 2.8) or *providing self-tests free-of-charge in screening centers* (m = 6.9, SD = 2.6).

### Disagreements

Although in cases where two expert groups brought up the same recommendation, they generally agreed on the level of importance they gave to this recommendation, a small number of these shared recommendations scored significantly differently from one expert group to the next. For example, *Self-tests available free-of-charge in screening centers*, *family planning centers*, *community organizations and services for substance users* (SD = 1.2) scored only 5.0 with MSM experts whereas their counterparts in the West Indies gave it 8.2, underlining the importance of diversity of access for different community groups.

Significant disagreement also occurred within individual expert groups on certain subjects. Discord levels were high within sub-Saharan migrant (m = 6.1, SD = 3.0) and Guiana (m = 4.4, SD = 3.4) expert groups concerning providing open access to self-tests for minors. One expert in the young people’s group explicitly stated that he was opposed to any access whatsoever to HIV self-testing for minors.

As stated above, the nature of the test itself proved to be the object of significant between-group disagreement: MSM experts explicitly favored self-testing using blood (m = 7.3), arguing that (a) blood tests would be perceived by the general public as being more reliable, (b) oral “saliva” testing would favor the ongoing false belief that HIV is to be found in the saliva, and (c) talking about “saliva” rather than “crevicular liquid” would be a sure source of errors. The young people’s experts, to the contrary, favored oral testing (m = 7.0), considering it to be more acceptable for the population in question.

## Discussion

The present study used the Delphi process with 72 experts on HIV screening and populations vulnerable to HIV to develop recommendations for good practice with regard to HIV self-testing in France. The main strength of the study was its scope, allowing a comparison of points of view of expert groups for eight different populations, including populations with both high and low HIV prevalence rates in France, and addressing a broad range of issues associated with providing access to self-testing.

Although a large number of recommendations were common to several groups of experts, the study highlighted a series of recommendations specific to each different population group, particularly with regard to information content and access both to information and to the self-tests themselves. The present results have thus already proved useful to health authorities at a national level in making recommendations and setting up standards for marketing self-tests in France. These issues ranged from accessing self-tests and accessing information and support on how to use self-tests in highly diverse community contexts, through to the key question of access to care for individuals facing positive self-test results]. In the present study for example, the recommendation that *the instructions must indicate what to do if you get a positive test result* was made by seven of the eight expert groups and received the highest overall mean score of recommendations common to three or more groups.

The sheer number of different recommendations made by the MSM experts compared to other expert groups in the present study is to be noted, potentially underlining the importance of HIV self-testing for this population group. For other groups, the same number of experts produced significantly lower numbers of different recommendations. This was particularly the case for the transgender experts who stressed the fact that HIV testing was often not a priority issue for this population group, being HIV positive being felt to be a barrier to accessing hormonal therapy or surgery. These experts insisted on the fact that, for transgender people to want to take care of themselves and look after their health, all barriers to the transitioning process needed to be removed.

The cost of self-tests is a significant issue, as has already been highlighted in several studies internationally. In 2012, in Singapore, with a screening center population amongst whom 40% earned less than US$1,500 per month, only 28% of subjects declared that they would accept to pay more than US$15 for a self-test [25]. This finding is in contrast with results published the same year in a Spanish study, where 40% of participants declared that they would be willing to pay €20 (US$23) or more [26]. In the present study, the recommendation that the self-test should be *at a moderate and accessible price for all* was brought up and validated by seven of the eight expert groups. However, less consensual was the proposition, debated in six groups, that self-tests should be *available free-of-charge in screening centers*, *family planning centers*, *community organizations*, *and services for people with substance abuse problems*. This recommendation scored highly for three groups who had also recommended *self-tests being free-of-charge for key populations with higher HIV risk* (sub-Saharan migrants, SU and FWI). However it was only given a mean score of 5.0 by the MSM expert group and 6.9 by the General Population and Young People experts. Clearly, the question of the price of self-tests is an issue with varying significance from one population group to the next. With regard to costs involved in accessing counselling and support, opinions were more consensual: four expert groups (sub-Saharan migrants, Trans, FWI and Young People) underlined the importance of there being *zero cost for the user to access telephone support services*, *including from smartphones*.

Another major issue was that, although self-testing is clearly a significant step towards user empowerment and sense of self-efficacy with regard to screening [27], experts in the present study insisted upon the need to provide access to information and support at each and every step of the self-testing process, underlining the importance of support and training for effective empowerment. In France, in the context of the recent government approval of HIV self-testing, the principle government-funded hotline providing information and support on HIV/AIDS free-of-charge and 24/7 will also ensure information and support for self-test users. However, linkage to counseling and care should the test result be positive is important, in concordance with recent findings from a New York study on MSM’s anticipated and actual reactions to obtaining positive self-test results [28]: subjects underlined not only the need to be able to access information, counselling and care, but also the question of handling their immediate emotional reaction when discovering a positive result and then dealing with potential stigma associated with being HIV positive.

The present study has a certain number of methodological limitations. First, the fact that only a limited number of experts accepted to participate in the French West Indies and Guiana expert groups clearly reduced the validity of results concerning these two populations. Seemingly, HIV was not amongst current priorities, no doubt in part due to the arrival of the chikungunya epidemic in the Caribbean area at the very moment the Delphi study was being launched. However, it is to be noted that (1) the four areas of expertise (research, screening and care, policy-making, community groups) were represented in both these expert groups and (2) a high proportion of recommendations were in common with the other expert groups. Second, the fact that all expert groups systematically included experts in HIV screening may have introduced a certain bias with regard to maintaining or defending existing screening services. Studies involving the different population groups in question with access to self-tests in real-life situations need to be undertaken to validate the findings derived from the experts participating in the present study. Third, the large number of recommendations and the fact that, for example, only one in three were common to three or more of the eight groups of experts, might well be a methodological artifact: indeed, researchers in the present study made a considerable effort to respect the different nuances expressed by each expert group. For example, the recommendation *Create a telephone hotline providing support on how to use the test*, *accessible 24/7*, common to all eight experts groups, was identified as a separate recommendation from that made by experts for young people and for substance users concerning providing a hotline specifically addressing support for users who discover positive self-test results: *Create a telephone hotline providing support in case of positive result*, *accessible 24/7*. Merging these separate but similar recommendations into one single recommendation might well have reduced the total number of recommendations and increased between-group concordance rates. In other cases, it is clear that on certain issues, in-depth interviews with experts might have provided more accurate and detailed recommendations. However, doing so many interviews would have been impossible given the importance of producing these recommendations before the HIV self-tests came onto the market in France. Furthermore, it must be underlined that, in the first round of the Delphi process, the experts were asked to give a short explanation with each of their initial recommendations. The researchers who analyzed the round one recommendations took all these comments into consideration when formulating the recommendations for round two of the Delphi process. Fourthly, in spite of the fact that the use of HIV self-tests in populations with low HIV prevalence like young people may lead to an increasing number of false positive test results, only two expert groups made recommendations concerning the reliability and performance of self-tests. This may well be due to the simple fact that this issue may have been seen by many experts as not being related to the principle research question concerning “good practices for responding to the information and support needs of HIV self-test users”. This should clearly not be interpreted as meaning that the other expert groups considered issues of specificity and sensitivity as not being important.

Finally, extrapolations from this French study to other national contexts are clearly problematic. The fact that the French health and social care system includes free access to HIV screening, treatment and care with no out-of-pocket payment will have inevitably influenced experts’ priorities in the present study.

## Conclusions

Results from the current study have already made a significant contribution to policy decisions concerning catering for the specific access, information and support needs of different potential HIV self-test user groups when these tests became publicly available in France in September 2015. Providing adapted access, information and support will contribute to facilitating screening both for people from high risk groups and for the general population, as well as potentially making an inroad into the hidden epidemic in France by bringing in vulnerable populations that had been reluctant to use standard testing options so far.

## Supporting Information

S1 File Recommendations with overall mean scores ≥7. This file details all recommendations with an overall mean score ≥7 on 9 according to these recommendations were:
common to at least three of the eight groups (Table A).made by the experts for men who have sex with men (MSM) (Table B).made by the experts for migrants from sub-Saharan Africa (Table C).made by the experts for substance users (SU) (Table D).made by the experts for transgender people (Table E).made by the experts for people living in the French West Indies (FWI) (Table F).made by the experts for people living in French Guiana (Table G).made by the experts for young people (<25) (Table H).made by the experts for the general population (Table I).
(PDF)
