# Supplementary material for: Comparing the Information and Support Needs of Different Population Groups in Preparation for 2015 Government Approval for HIV Self-testing in France
Source: PLoS One. 2016 Mar 31;11(3):e0152567. doi: 10.1371/journal.pone.0152567 (PMC4816498; doi:10.1371/journal.pone.0152567)
Supplement: S1 File — This file details all recommendations with an overall mean score ≥7 on 9 according to these recommendations were: common to at least three of the eight groups (Table A).made by the experts for men who have sex with men (MSM) (Table B).made by the experts for migrants from sub-Saharan Africa (Table C).made by the experts for substance users (SU) (Table D).made by the experts for transgender people (Table E).made by the experts for people living in the French West Indies (FWI) (Table F).made by the experts for people living in French Guiana (Table G).made by the experts for young people (<25) (Table H).made by the experts for the general population (Table I). (PDF) [file pone.0152567.s001.pdf]

Recommendations for HIV Self-testing in France. Greacen et al.

Supporting information: S1 File

Recommendations with overall mean scores ≥7

This file details all recommendations with an overall mean score ≥7 on 9 according to these recommendations were:

- common to at least three of the eight groups (Table A)
- made by the experts for men who have sex with men (Table B)
- made by the experts for migrants from sub-Saharan Africa (Table C)
- made by the experts for substance users (Table D)
- made by the experts for transgender people (Table E)
- made by the experts for people living in the French West (Table F)
- made by the experts for people living in French Guiana (Table G)
- made by the experts for young people (<25) (Table H)
- made by the experts for the general population (Table I)

Table A: Recommendations with overall mean scores ≥7 common to at least three of the eight groups, presented by decreasing overall mean scores and including mean scores for each expert group.

Recommendations	N groups	Mean	SD	MSM	MiSSA	SU	Trans	FWI	Guia na	Young	Gen Pop
The instructions must indicate what to do if you get a positive test result.	7	8.9	0.1	8.9	8.9	8.6		9.0	9.0	9.0	8.9
The instructions on how to use the self-test and how to interpret test results need to be clear and comprehensible for all users.	8	8.6	0.3	8.9	8.9	8.2	8.3	9.0	8.6	8.5	8.6
The instructions need to indicate how to read test results, including results that are unclear or invalid.	3	8.5	0.2	8.4						8.7	8.5
A support service that can direct the person who gets a positive test result to a clearly identified care and support network.	6	8.4	0.4	8.4	8.1	8.4	8.0	9.0		8.3	
The instructions include information on how to access 24/7 free-of-charge telephone support on using the self-test, similar to that currently provided by Sida Info Service[1].	5	8.4	0.6	8.8	8.9	7.7				8.9	7.9
Access to self-tests at a reasonable price or free-of-charge for population groups with greater HIV risk.	3	8.4	0.4		8.2	8.8		8.1			
The instructions should underline the importance of knowing if you are HIV positive as early as possible An optimistic approach to HIV care and living with HIV.	4	8.3	0.7		9.0	7.7		8.8		7.8	
The instructions should indicate how to interpret a negative result and understand the implications of testing during the seroconversion window period.	8	8.2	0.5	8.1	8.1	8.0	8.6	8.5	9.0	8.5	7.5

A moderate and accessible price for all.	7	8.2	0.6	8.6	8.2	8.8	7.7	9.0		7.3	7.7
A test that is easy to use.	4	8.2	0.6	7.5	8.8				8.6		7.8
The instructions should use pictograms.	7	8.1	0.6	7.9	8.5	7.4	8.6	8.8		8.2	7.1
Evaluate self-test usage.	4	8.1	0.6	7.8				8.3		8.8	7.5
The instructions should be sufficiently concise to be easily readable in one sitting.	3	8.1	0.5		7.5			8.3	8.4		
Public information concerning self-tests should be part of a general campaign promoting HIV screening and situating self-tests as one of a number of available test options, all of which have their specific advantages.	5	7.9	0.4	8.0				8.3	8.0	8.2	7.2
The self-test helpline should have personnel specifically trained with regard to the different population groups with higher HIV prevalence.	3	7.8	0.2	7.7		8.1			7.7		
Underline the importance of only using self-tests that are approved for use in France.	4	7.8	0.2	8.1	7.6			7.7		7.8	
Prepare and train those who will be selling or distributing self-tests (drugstores, community organizations…) or who provide support in using self-tests.	6	7.8	0.6	6.6	8.2	7.8		8.2	7.7	8.3	
The instructions should address the question of the need for support if a test result is positive: who should I speak to? Listing the different possible sources for accessing support.	4	7.8	0.6			6.9		8.0		8.3	7.9
Zero cost for the user to access telephone support services, including from smartphones.	4	7.8	0.6		7.5		7.4	8.7		7.5	
The instructions need to explain that saliva cannot transmit the virus, even if you can diagnose HIV using saliva.	4	7.8	0.8		8.7	6.9		7.5		8.2	
Create a telephone hotline providing support on how to use the test, accessible 24/7.	8	7.8	0.9	8.9	7.7	6.8	7.9	8.7	6.2	8.5	7.7
The instructions should include contact details on how to access community support or healthcare at a local level.	5	7.8	1.0	6.5	8.1	7.0		9.0	8.3		
Situate self-tests as a complementary strategy with regard to existing screening options.	6	7.7	0.5	7.9	7.7			7.7	8.4	7.9	6.8
Provide a variety of different ways of accessing self-tests, taking into account cultural and geographical diversity and in terms of cost.	6	7.7	1.0	6.1	7.7	7.4	8.6		8.8	7.4	
Information needs to be available in the different foreign languages spoken in France, including in the overseas French departments.	4	7.7	1.0		8.0	7.7	8.8				6.3
Before the self-test comes onto the market, prepare (train, equip) STI screening enters, HIV community organisations, and professionals working with key populations.	3	7.7	1.0	8.3		8.2		6.5			
An online self-test assistance service (chat / SMS), 24/7.	3	7.6	0.9					6.6	7.9		8.4

The instructions must underline the importance of continuing to protect yourself, using condoms and sterile equipment.	4	7.6	0.8	6.8				8.3		8.1	7.0
The instructions should be culturally adapted to the different communities affected by HIV and validated with these communities.	3	7.6	0.8	8.2	6.7						7.8
The instructions should orientate users to an Internet site where they can see videos of the test being used.	4	7.5	0.3	7.6	7.8	7.6				7.0	
Access via Internet.	3	7.5	1.3		8.4			6.0	8.0		
Self-tests should be widely accessible, not just in drugstores.	6	7.3	0.8	7.7		7.2		8.2	7.8	6.5	6.2
Communicate with health professionals and community organizations ahead of communicating with the general public. Make health professionals aware of how the self-test will contribute to individuals' prevention strategies, its position with regard to other screening options and how best to access care if test results are positive.	5	7.3	0.7			7.8		7.5	7.7	7.5	6.2
Self-test outlets, including sales outlets (drugstores, etc.) must be able to provide oral information by personnel trained to inform and assist with self-test use.	4	7.2	0.9		7.7			7.7	5.8	7.5	
Self-tests available free-of-charge in screening centers, family planning centers, community organizations, and services for people with substance abuse problems.	6	7.2	1.2	5.0	8.0	8.2		8.2		6.9	6.9
The instructions need to emphasize that a negative test result does not mean that your partner is negative, even if you had unprotected sex.	3	7.2	0.5					6.6	7.4		7.5
Conduct specific campaigns promoting the self-test with the different high-risk population groups and taking into account their specificities.	7	7.1	0.6	7.1	7.7	6.5	7.3		8.2	6.7	6.5
Find ways of talking about HIV without being stigmatizing. Give users the words they will need to dare talk about it.	3	7.1	0.1	7.1			7.1			7.2	
Prepare your self-testing (choose the right place, time of day, entourage, state of mind).	3	7.1	0.7		6.3		7.7		7.2		
The instructions should include general information on HIV, HIV transmission and prevention.	5	7.0	1.2		7.0	5.0		8.2	7.4	7.6	
Inform users about the reliability of self-tests (sensitivity and specificity).	3	7.0	1.6						8.1	5.1	7.7

[1] Sida Info Service: AIDS Info Service, a government-funded hotline providing information and support on HIV/AIDS in France

SD: standard deviation; MSM: men who have sex with men; MiSSA: migrants from sub-Saharan Africa; SU: substance users; Trans: transgender people; FWI: French West Indies; Guiana: French Guiana; Young: young people; Gen Pop: general population.

Table B: Shared and specific recommendations made by the experts for men who have sex with men (MSM) and scoring ≥7

Recommendations	Mean	SD	Other groups that considered this reco.[1]
Create a telephone hotline providing support on how to use the test, accessible 24/7.	8.9	0.3	≥3
The instructions on how to use the self-test and how to interpret test results need to be clear and comprehensible for all users.	8.9	0.3	≥3
The instructions must indicate what to do if you get a positive test result.	8.9	0.3	≥3
Give preference to the most reliable tests (in terms of sensitivity and specificity).	8.9	0.3	Gen Pop 7.5
The instructions include information on how to access 24/7 free-of-charge telephone support on using the self-test, similar to that currently provided by Sida Info Service[2].	8.8	0.4	≥3
Access in drugstores and in other stores selling health products.	8.7	0.5	SU 8.1
Test results that are easy to understand.	8.7	0.5	MiSSA 8.1
An online/telephone support service run by an organization with experience in informing people about HIV.	8.7	0.5	Gen Pop 5.8
Open access, not just on prescription.	8.7	0.7	SU 8.7
A moderate and accessible price for all.	8.6	0.7	≥3
A support service that can direct the person who gets a positive test result to a clearly identified care and support network.	8.4	0.7	≥3
The instructions should be sufficiently concise to be easily readable in one sitting.	8.4	0.8	≥3
The instructions need to indicate how to read test results, including results that are unclear or invalid.	8.4	0.8	≥3
Over-the-counter access for the general public at a price no higher than a standard HIV test prescribed by a doctor.	8.4	1.0	0
The instructions need to say what to do if you get a test result that is not clear or invalid.	8.4	1.0	0
A support service run by an independent organization, e.g. Sida Info Service[2].	8.3	1.3	FWI 8.8
For population groups such as MSM with high prevalence rates and multiple risk-taking, stress the importance of doing the test regularly, of repeating the test; and place less focus on waiting until the end of the seroconversion window after taking a risk.	8.2	1.2	0
Inform users about the reliability of self-tests (sensitivity and specificity).	8.1	0.7	≥3
A wide reaching campaign promoting the support service (such as Sida Info Service[2] or suchlike in France).	8.1	1.1	0
Create and use a public communication platform to inform about self-testing, and not rely on a private platform developed by the self-test manufacturer.	8.1	1.3	SU 7.4
Underline the importance of only using self-tests that are approved for use in France.	8.1	1.4	≥3
The instructions should indicate how to interpret a negative result and understand the implications of testing during the seroconversion window period.	8.1	1.9	≥3
Public information concerning self-tests should be part of a general campaign promoting HIV screening and situating self-tests as one of a number of available test options, all of which have their specific advantages.	8.0	0.9	≥3
Inform users about early HIV acute infection symptoms and the greater risk of transmitting the HIV virus during this phase.	8.0	1.1	0

Access via internet.	8.0	1.6	≥3
An online self-test assistance service (chat / SMS), 24/7.	7.9	0.9	≥3
Situate self-tests as a complementary strategy with regard to existing screening options.	7.9	1.1	≥3
The instructions should use pictograms.	7.9	1.3	≥3
Add questions about HIV self-tests in existing surveys.	7.9	1.8	0
Put into place an accredited training program for telephone support service staff.	7.8	1.2	0
Review marketing approval regularly in order to guarantee self-test quality.	7.8	1.4	0
Evaluate self-test usage.	7.8	2.1	≥3
Support service staff need to receive specific training regarding the community groups that are more vulnerable with regard to HIV.	7.7	1.3	0
Self-tests should be widely accessible, not just in drugstores.	7.7	1.6	≥3
The instructions need to appear sufficiently attractive so that even people who think they already know how to use the self-test will read them.	7.6	1.0	Young 8.4
The instructions should cover the subject of how regularly people with different risk-taking frequencies should do the test. Doing the test frequently, repeating the test, are important for community contexts with high HIV prevalence and frequent risk-taking.	7.6	1.3	0
The instructions should include a QR code or URL linking to a website presenting a film providing visual instructions on how to use the test.	7.6	1.5	0
A test that is easy to use.	7.5	1.4	≥3
Inform users about other STIs, hepatitis, etc.	7.5	1.4	FWI 8.3
The instructions need to emphasize that a negative test result does not mean that your partner is negative, even if you had unprotected sex.	7.4	1.4	≥3
Use HIV self-testing to promote screening for other STIs in vulnerable populations.	7.3	1.5	Trans 7.6
Information for MSM should focus less on high risk behavior, and more on their higher risk epidemiological context (high prevalence of HIV in this population).	7.3	1.6	0
Give preference to self-tests using blood samples.	7.3	2.1	0
Inform people about the things they should automatically consider doing after having unsafe sex.	7.2	1.6	0
Prepare your self-testing (choose the right place, time of day, entourage, state of mind).	7.2	2.0	≥3
Neutral packaging guaranteeing confidentiality and anonymity if being sent by post.	7.2	2.1	0
The instructions should inform users about their rights and responsibilities with regard to HIV self-testing.	7.1	1.9	≥3
Conduct specific campaigns promoting the self-test with the different high-risk population groups and taking into account their specificities.	7.1	2.1	≥3
Guarantee reagent tracing.	7.1	2.1	0
The instructions should suggest to the user that, before doing the test, they should think about who they could talk to if the test result were positive.	7.1	2.5	0
Inform users about HIV treatment and care.	7.0	1.6	0
Use the campaign promoting self-tests for the general public to get messages through to hidden vulnerable population groups for whom	7.0	1.8	Gen Pop 5.8

anonymity might be a crucial issue.			
Self-test promotion needs to remind people about the types of sexual relations which are at risk with regard to HIV.	7.0	1.9	Trans 7.6

[1]A zero (0) indicates a recommendation that is specific to this population. In cases in which the recommendation is common to only one other expert group, this group is indicated with its mean score. "≥3" means that this recommendation was brought up by two or more other groups of experts (amongst these recommendations brought up by ≥3 groups, those with an overall mean score ≥7 are also presented in Table S1).

[2]Sida Info Service: AIDS Info Service, a government-funded hotline providing information and support on HIV/AIDS in France

SD: standard deviation; MSM: men who have sex with men; MiSSA: migrants from sub-Saharan Africa; SU: substance users; Trans: transgender people; FWI: French West Indies; Guiana: French Guiana; Young: young people; Gen Pop: general population.

Table C: Shared and specific recommendations made by the experts for migrants from Sub-Saharan Africa (MiSSA) and scoring ≥7

Recommendations	Mean	SD	Other groups that considered this reco.[1]
A single-use test.	9.0	0.0	0
The instructions should underline the importance of knowing if you are HIV positive as early as possible. An optimistic approach to HIV care and living with HIV.	9.0	0.0	0
Test results that are easy to understand.	9.0	0.0	MSM 8.7
The instructions on how to use the self-test and how to interpret test results need to be clear and comprehensible for all users, including those who do not have a high educational level.	8.9	0.3	≥3
The instructions include information on how to access 24/7 free-of-charge telephone support on using the self-test, similar to that currently provided by Sida Info Service.[2]	8.9	0.3	≥3
The instructions must indicate what to do if you get a positive test result.	8.9	0.3	≥3
A test that is easy to use.	8.8	0.4	≥3
The instructions need to explain that saliva cannot transmit the virus, even if you can diagnose HIV using saliva.	8.7	0.5	≥3
Use the public self-test training and information website to collect users' accounts concerning the difficulties in using or understanding how to use the test, and any particularly useful or helpful points'.	8.5	0.7	0
The instructions should use pictograms.	8.5	1.0	≥3
A test with no risk that third parties might get contaminated.	8.5	1.3	0
The instructions should remind users that self-testing is just one of a series of possible ways of accessing screening and preventing HIV transmission.	8.4	1.0	0
Before the self-test comes onto the market, prepare (train, equip) STI screening enters, HIV community organisations, and professionals working with key populations.	8.3	0.9	≥3
Consolidate working relationships between community organizations and HIV screening and healthcare services.	8.3	0.9	0
The instructions need to make it clear that, in France, HIV care is financed entirely by the government, no matter you're your administrative status might be (i.e. whether you are a French citizen, a legal resident, or even an illegal immigrant).	8.3	1.3	0

A moderate and accessible price for all.	8.2	0.8	≥3
Prepare and train those who will be selling or distributing self-tests (drugstores, community organizations…) or who provide support in using self-tests.	8.2	0.9	≥3
The instructions should be culturally adapted to the different communities affected by HIV and validated with these communities.	8.2	0.9	≥3
Access free-of-charge for less privileged populations.	8.2	1.6	0
A support service that can direct the person who gets a positive test result to a clearly identified care and support network.	8.1	1.1	≥3
The instructions should include contact details on how to access community support or healthcare at a local level.	8.1	1.2	≥3
The instructions should indicate how to interpret a negative result and understand the implications of testing during the seroconversion window period.	8.1	1.6	≥3
Information needs to be available in the different foreign languages spoken in France, including in the overseas French departments.	8.0	1.1	≥3
Self-tests available free-of-charge in screening centers, family planning centers, community organizations, and services for people with substance abuse problems.	8.0	2.5	≥3
Fix distribution and sales rules that guarantee sales only to individuals or bulk supply to legitimate organizations (public services, community NGOs, etc.).	7.9	1.2	0
Monitor self-test use via community organizations.	7.9	1.3	0
Organize the publicity campaign taking into account the different population groups that are more highly concerned by HIV.	7.9	1.3	0
The instructions should orientate users to an internet site where they can see videos of the test being used.	7.8	1.7	≥3
People selling or distributing self-tests need to do so in a positive way.	7.7	0.9	0
Conduct specific campaigns promoting the self-test with the different high-risk population groups and taking into account their specificities.	7.7	1.1	≥3
Use communications media targeting the vulnerable population groups at the same time as the general public media when communicating about the arrival of the self-test.	7.7	1.3	≥3
Provide a variety of different ways of accessing self-tests, taking into account cultural and geographical diversity and in terms of cost.	7.7	1.3	≥3
Situate self-tests as a complementary strategy with regard to existing screening options.	7.7	1.4	≥3
The telephone support service staff need to receive specific training on the community population groups that are more vulnerable to HIV.	7.7	1.6	≥3
Self-test outlets, including sales outlets (drugstores, etc.) must be able to provide oral information by personnel trained to inform and assist with self-test use.	7.7	1.7	≥3
Create a telephone hotline providing support on how to use the test, accessible 24/7.	7.7	1.8	≥3
The instructions must indicate storage conditions for the self-test.	7.7	2.1	FWI 7.5
Underline the importance of only using self-tests that are approved for use in France.	7.6	1.8	≥3
Use communication tools that are adapted for this population group (shape, color, etc…), i.e. specific tools.	7.5	1.4	0
Zero cost for the user to access telephone support services, including from smartphones.	7.5	2.2	≥3

When handing out/distributing the self-test inform the person about the possibility of doing a rapid HIV test (either on site or send them to a place where they can get a rapid HIV test), with pre- and post-test counselling.	7.4	1.4	0
The instructions should provide complementary information on STIs and viral hepatitis, and how to screen for them, emphasizing the fact that they cannot be detected by the HIV self-test.	7.3	1.8	≥3
Regulations need to ensure over-the-counter sales, the only way to guarantee efficient distribution.	7.2	2.0	0
The instructions should inform users about their rights and responsibilities with regard to HIV self-testing.	7.2	2.3	≥3
Use any and all points of contact between vulnerable populations and the health and social care system to provide information about self-testing.	7.1	1.3	SU 7.9
Find ways of talking about HIV without being stigmatizing. Give users the words they will need to dare talk about it.	7.1	1.4	≥3
Identify places where potential users can access training on how to use self-tests.	7.1	1.5	0
In addition to the 24/7 hotline, when delivering the self-test ensure distribution of the phone numbers of the main community organizations.	7.1	1.7	0
Do not create a specific self-test access route for migrants; rather create specific support strategies for migrants in vulnerable situations.	7.1	1.7	0
The instructions need to remind people about and explain post-exposure treatments.	7.1	1.7	0
A telephone support service with access to professional interpreters.	7.1	2.0	0
The instructions should include general information on HIV, HIV transmission and prevention.	7.0	1.5	≥3

[1]A zero (0) indicates a recommendation that is specific to this population. In cases in which the recommendation is common to only one other expert group, this group is indicated with its mean score. "≥3" means that this recommendation was brought up by two or more other groups of experts (amongst these recommendations brought up by ≥3 groups, those with an overall mean score ≥7 are also presented in Table S1).

[2]Sida Info Service: AIDS Info Service, a government-funded hotline providing information and support on HIV/AIDS in France

SD: standard deviation; MSM: men who have sex with men; MiSSA: migrants from sub-Saharan Africa; SU: substance users; Trans: transgender people; FWI: French West Indies; Guiana: French Guiana; Young: young people; Gen Pop: general population.

Table D: Shared and specific recommendations made by the experts for substance users (SU) and scoring ≥7

**Recommendations**	**Mean**	**SD**	**Other groups that considered this reco.**[1]
A moderate and accessible price for all.	8.8	0.4	≥3
Open access, not just on prescription.	8.7	0.6	MSM 8.7
The instructions must indicate what to do if you get a positive test result.	8.6	0.7	≥3
Work together with the substance user community organizations to develop the tools needed for making substance users aware of the possibility of doing self-tests and for setting up ways of accessing self-tests and appropriate support.	8.5	0.7	MiSSA 6.9[3]
Train all stakeholders working with substance users on promoting the self-test and explaining how to use it.	8.5	0.9	0
Access via internet.	8.4	0.8	≥3

A support service that can direct the person who gets a positive test result to a clearly identified care and support network.	8.4	0.8	≥3
Accessible everywhere geographically.	8.4	1.6	Guiana 7.8
Inform substance users about care services where a confirmatory test can be done.	8.3	1.0	0
Use the community approach.	8.2	0.8	MiSSA 6.0
The instructions on how to use the self-test and how to interpret test results need to be clear and comprehensible for all users, including those who do not have a high educational level.	8.2	0.9	≥3
Provide access to self-tests free of charge in substance use care services.	8.2	2.4	≥3
Access to self-tests at a reasonable price or free-of-charge for population groups with greater HIV risk.	8.2	2.4	≥3
Identify and prepare HIV care access points in case of positive results.	8.1	0.8	FWI 8.8
Access in drugstores and in other stores selling health products.	8.1	1.2	MSM 8.7
The instructions should indicate how to interpret a negative result and understand the implications of testing during the seroconversion window period.	8.0	0.9	≥3
Use any and all points of contact between substance users and the health and social care system to provide information about self-testing.	7.9	0.9	MiSSA 7.1
Use communications media targeting the vulnerable population groups at the same time as the general public media when communicating about the arrival of the self-test.	7.8	1.0	≥3
Prepare and train those who will be selling or distributing self-tests (drugstores, community organizations…) or who provide support in using self-tests.	7.8	1.2	≥3
Prepare and train professionals working with substance users on providing support and counselling to users whose self-test result is positive.	7.8	1.4	0
Communicate about the self-test to professional and community organizations before communicating to the general public. Make healthcare stakeholders aware of the added value of self-testing with regard to individual prevention strategies, of its place in the screening system and access to care in the event of positive results.	7.8	1.5	≥3
The instructions need to make it clear that all self-test results, whether they are positive or negative, need to be confirmed by a conventional test.	7.8	2.4	0
Information needs to be available in the different foreign languages spoken in France, including in the overseas French departments.	7.7	1.3	≥3
The instructions should underline the importance of knowing if you are HIV positive as early as possible An optimistic approach to HIV care and living with HIV.	7.7	1.4	≥3
The instructions include information on how to access 24/7 free-of-charge telephone support on using the self-test, similar to that currently provided by Sida Info Service[2].	7.7	1.8	≥3
Access to self-tests and support on self-testing in all places that are in contact with substance users.	7.7	2.3	0
The instructions should orientate users to an internet site where they can see videos of the test being used.	7.6	1.3	≥3
Make sure that self-tests are accessible for substance users who don't access other forms of testing.	7.6	2.4	0
Identify best practice support and counselling criteria.	7.5	0.9	0
The instructions should be sufficiently concise to be easily readable in one sitting.	7.5	2.0	≥3

Propose sending self-tests to substance users in the same way that substance user services send harm reduction material via the post.	7.5	2.1	0
Create and use a public communication platform for self-tests, and not rely on a private platform developed by the self-test manufacturer.	7.4	1.8	MSM 8.1
The instructions should use pictograms.	7.4	1.9	≥3
Provide a variety of different ways of accessing self-tests, taking into account cultural and geographical diversity.	7.4	3.0	≥3
Publicize self-tests on injection kit packaging or other harm-reduction tools.	7.3	1.7	0
For confirmatory screening, identify screening centers that know how to look after injecting substance users who would be mistrustful of a blood sample being taken by someone else.	7.2	2.4	0
Self-tests should be widely accessible, not just in drugstores.	7.2	3.0	≥3
The instructions should include contact details on how to access community support or healthcare at a local level.	7.0	1.8	≥3

[1]A zero (0) indicates a recommendation that is specific to this population. In cases in which the recommendation is common to only one other expert group, this group is indicated with its mean score. "≥3" means that this recommendation was brought up by two or more other groups of experts (amongst these recommendations brought up by ≥3 groups, those with an overall mean score ≥7 are also presented in Table S1).

[2]Sida Info Service: AIDS Info Service, a government-funded hotline providing information and support on HIV/AIDS in France

[3]For the MiSSA expert group, this recommendation was formulated as follows: Work together with the community organizations of key populations to develop the tools needed for making potential users aware of the possibility of doing self-tests and for setting up ways of accessing self-tests and appropriate support.

SD: standard deviation; MSM: men who have sex with men; MiSSA: migrants from sub-Saharan Africa; SU: substance users; Trans: transgender people; FWI: French West Indies; Guiana: French Guiana; Young: young people; Gen Pop: general population.

Table E: Shared and specific recommendations made by the experts for transgender people and scoring ≥7

**Recommendations**	**Mean**	**SD**	**Other groups that considered this reco.[1]**
Evaluate linkage to care for people who discover positive test results using self-tests.	8.9	0.3	Gen Pop 7.7
Have self-tests available free-of-charge in community organizations working with sex workers.	8.8	0.4	0
Information needs to be available in the different foreign languages spoken by the key populations.	8.8	0.4	≥3
Access to self-tests at a reasonable price or free-of-charge for population groups with greater HIV risk.	8.8	0.7	≥3
Train online support staff on the specific problems of the different transgender communities.	8.8	0.7	0
In community organizations and places that transgender people frequent, propose training on how to use the self-test and on your rights with regard to self-testing.	8.7	0.5	0
The instructions should use pictograms.	8.6	0.5	≥3
The instructions should indicate how to interpret a negative result and understand the implications of testing during the seroconversion window period.	8.6	0.7	≥3
Provide a variety of different ways of accessing self-tests [for population groups that are vulnerable to HIV], taking into account cultural and geographical diversity and in terms of cost.	8.6	1.0	≥3

A support service also available in foreign languages.	8.4	0.5	Guiana 7.4
The instructions on how to use the self-test and how to interpret test results need to be clear and comprehensible for all users, including those who do not have a high educational level.	8.3	1.3	≥3
Before the self-test comes onto the market, prepare (train, equip) STI screening enters, HIV community organisations, and professionals working with key populations.	8.2	0.8	≥3
Train peer trainers amongst the populations most affected by HIV.	8.2	1.4	0
The self-test helpline should have personnel specifically trained with regard to the different population groups with higher HIV prevalence.	8.1	0.9	≥3
Recall peoples' rights and responsibilities with regard to HIV self-testing in a specific legal text.	8.1	0.9	≥3[2]
In communicating, don't forget female-to-male transgender people.	8.1	1.3	0
Create a community medical network for Transgender people, so that they can consult someone after doing a self-test, no matter what the result was.	8.1	1.5	0
A support service that knows how to help different population groups get their test results confirmed or access care, and particularly so for populations who don't have an easy relationship with the care system (transgender, substance users) or who have no established relationship at all with the care system (e.g. young people).	8.0	0.9	0
Respect peoples' genders.	8.0	1.9	0
Create a telephone hotline providing support on how to use the test, accessible 24/7.	7.9	1.7	≥3
Make sure not to put identities, life experiences or sexualities into any sort of hierarchical order.	7.9	1.7	0
Propose the option of having someone else being there while the person uses the self-test and when they get the results.	7.8	1.3	0
Encourage self-test users to promote self-test use amongst their partners. Promote the image of the self-test user as a stakeholder with an active role in community health.	7.7	0.9	0
A moderate and accessible price for all.	7.7	2.6	≥3
Self-test promotion needs to remind people about the types of sexual relations which are at risk with regard to HIV.	7.6	1.0	MSM 7.0
The instructions should inform users about their rights and responsibilities with regard to HIV self-testing.	7.6	1.1	≥3
Promote user autonomy and empowerment.	7.6	1.2	Young 6.0
Demedicalize communication addressing transgender people.	7.6	1.4	0
Use HIV self-testing to promote screening for other STIs in vulnerable populations.	7.6	2.0	MSM 7.3
When communicating about the self-test, remember that certain transgender groups use the free and anonymous screening centers (French CDAG/CIDDIST) as a point of contact with the healthcare system.	7.4	0.9	0
The instructions should suggest anticipating a positive result.	7.4	1.4	0
As soon as possible, introduce combination self-tests that will do HIV and other STIs at the same time.	7.4	1.4	0
Zero cost for the user to access telephone support services, including from smartphones.	7.4	1.8	≥3
Make it possible to sell several tests in the same packet.	7.4	2.0	MSM 6.0

Use transgender-targeting communications media at the same time as the general public media when communicating about the arrival of the self-test.	7.3	1.5	0
Conduct specific campaigns promoting the self-test with the different high-risk population groups and taking into account their specificities.	7.3	2.1	≥3
Access to care that does not interrupt hormone therapy.	7.2	2.5	0
Remove administrative barriers, favour the right to hormonal therapy and to surgery to enable transgender people to take care of themselves better and facilitate screening.	7.2	2.7	0
Respect the diversity of transgender people: fight against conventional stereotyping.	7.1	1.5	0
Do not over « medicalize » self-test instructions.	7.0	1.0	Guiana 8.0
Involve not only NGOs but also informal groups.	7.0	1.3	0
The instructions must list likely interactions with substances or medication being used, mouth and teeth problems, hormonal treatment or hormone cycles and, conversely, reassure users if there are unlikely to be any problematic interactions.	7.0	1.5	SU 4.5

[1]A zero (0) indicates a recommendation that is specific to this population. In cases in which the recommendation is common to only one other expert group, this group is indicated with its mean score. "≥3" means that this recommendation was brought up by two or more other groups of experts (amongst these recommendations brought up by ≥3 groups, those with an overall mean score ≥7 are also presented in Table S1).

[2]Overall mean score = 6.9. Other groups who considered this item: MiSSA (6.6) and Gen pop (5.5)

SD: standard deviation; MSM: men who have sex with men; MiSSA: migrants from sub-Saharan Africa; SU: substance users; Trans: transgender people; FWI: French West Indies; Guiana: French Guiana; Young: young people; Gen Pop: general population.

Table F: Shared and specific recommendations made by the experts for people living in the French West Indies (FWI) and scoring ≥7

**Recommendations**	**Mean**	**SD**	**Other groups that considered this reco.[1]**
The instructions on how to use the self-test and how to interpret test results need to be clear and comprehensible for all users, including those who do not have a high educational level.	9.0	0.0	≥3
The instructions should include contact details on how to access community support or healthcare at a local level.	9.0	0.0	≥3
The instructions must indicate what to do if you get a positive test result.	9.0	0.0	≥3
A moderate and accessible price for all.	9.0	0.0	≥3
A support service that can direct the person who gets a positive test result to a clearly identified care and support network.	9.0	0.0	≥3
Identify people / organizations that are accessible 24/7 to listen to and counsel people who get positive test results.	9.0	0.0	0
Identify and prepare HIV care access points in case of positive results.	8.8	0.4	SU 8.1
The instructions should use pictograms.	8.8	0.4	≥3
The instructions should underline the importance of knowing if you are HIV positive as early as possible An optimistic approach to HIV care and living with HIV.	8.8	0.4	≥3
Ensure support as much for people who get negative results as for those who get positive results.	8.8	0.4	Young 7.7

A telephone support service provided by an « independent » organization like Sida Info Service[2].	8.8	0.4	MSM 8.3
Create a telephone hotline providing support on how to use the test, accessible 24/7.	8.7	0.5	≥3
Zero cost for the user to access telephone support services, including from smartphones.	8.7	0.5	≥3
The instructions must explain the implications of the window period preceding HIV seroconversion.	8.7	0.8	Guiana 8.6
The instructions should indicate how to interpret a negative result and understand the implications of testing during the seroconversion window period.	8.5	1.2	≥3
The instructions must be available in the foreign languages spoken in mainland France and in the French Overseas Departments (Guiana, Guadeloupe, Martinique, Reunion Island, Mayotte…).	8.3	1.0	Guiana 8.2
Evaluate self-test usage.	8.3	1.0	≥3
Specify the reliability of the different self-tests (oral, blood).	8.3	1.2	Gen Pop 4.7
Inform users about other STIs, hepatitis, etc.	8.3	1.2	MSM 7.5
The instructions must underline the importance of continuing to protect yourself, using condoms and sterile equipment.	8.3	1.2	≥3
Public information concerning self-tests should be part of a general campaign promoting HIV screening and situating self-tests as one of a number of available test options, all of which have their specific advantages.	8.3	1.6	≥3
Self-tests available free-of-charge in screening centers, family planning centers, community organizations, and services for people with substance abuse problems.	8.2	0.8	≥3
Prepare and train those who will be selling or distributing self-tests (drugstores, community organizations…) or who provide support in using self-tests.	8.2	1.0	≥3
The instructions should include general information about HIV, HIV transmission and prevention.	8.2	1.0	≥3
Self-tests should be widely accessible, not just in drugstores.	8.2	1.0	≥3
The instructions must make it clear that the self-test is a single-use test.	8.2	1.2	0
Favour wide-scale access, outside healthcare centers, including vouchers to allow people to obtain self-tests for free in drugstores.	8.2	1.2	0
State people's rights and responsibilities with regard to HIV self-testing.	8.0	0.6	Guiana 7.0
Emphasize the reliability of the self-test, if used correctly.	8.0	0.9	Young 6.9
The instructions should address the question of the need for support if a test result is positive: who should I speak to? Listing the different possible sources for accessing support.	8.0	0.9	≥3
The instructions should inform all stakeholders (health system, community organizations, public authorities, general public…) about their rights and responsibilities with regard to HIV self-testing.	8.0	1.1	≥3
The instructions should inform self-test users about their rights and responsibilities with regard to self-testing.	8.0	1.5	0
Self-tests at a low price or free of charge in healthcare centers.	7.8	1.2	Guiana 6.2
Clear information on the limits of the self-test and on how hard it might be finding oneself all alone with a positive result.	7.8	1.9	0

The instructions should provide complementary information on STIs and viral hepatitis, and how to screen for them, emphasizing the fact that they cannot be detected by the HIV self-test.	7.8	1.9	≥3
Insist upon being able to access the self-test without having to go through a health professional.	7.7	0.8	0
Prepare your self-testing (choose the right place, time of day, entourage, state of mind).	7.7	1.4	≥3
Underline the importance of only using self-tests that are approved for use in France.	7.7	1.5	≥3
Give preference to distribution circuits that protect people's confidentiality, for example using dispensers.	7.7	1.5	≥3[3]
Self-test outlets, including sales outlets (drugstores, etc.) must be able to provide oral information by personnel trained to inform and assist with self-test use.	7.7	1.9	≥3
Situate self-tests as a complementary strategy with regard to existing screening options.	7.7	2.3	≥3
The instructions must indicate storage conditions for the self-test.	7.5	1.0	MiSSA 7.7
Communicate about the self-test to professional and community organizations before communicating to the general public. Make healthcare stakeholders aware of the added value of self-testing with regard to individual prevention strategies, of its place in the screening system and access to care in the event of positive results.	7.5	1.4	≥3
The instructions should explain that you cannot catch HIV from saliva, even if the diagnosis of being infected with HIV can be made using saliva.	7.5	2.3	≥3
Find ways of talking about HIV without being stigmatizing. Give users the words they will need to dare talk about it.	7.1	1.8	≥3

[1]A zero (0) indicates a recommendation that is specific to this population. In cases in which the recommendation is common to only one other expert group, this group is indicated with its mean score. "≥3" means that this recommendation was brought up by two or more other groups of experts (amongst these recommendations brought up by ≥3 groups, those with an overall mean score ≥7 are also presented in Table S1).

[2]Sida Info Service: AIDS Info Service, a government-funded hotline providing information and support on HIV/AIDS in France

[3]Overall mean score = 6.1. Other groups who considered this item: MSM (5.0) and Young (5.6)

SD: standard deviation; MSM: men who have se≥3 with men; MiSSA: migrants from sub-Saharan Africa; SU: substance users; Trans: transgender people; FWI: French West Indies; Guiana: French Guiana; Young: young people; Gen Pop: general population.

Table G: Shared and specific recommendations made by the ≥3 experts for people living in French Guiana and scoring ≥7

**Recommendations**	**Mean**	**SD**	**Other groups that considered this reco.[1]**
The instructions should indicate how to interpret a negative result and understand the implications of testing during the seroconversion window period.	9.0	0.0	≥3
The instructions must indicate what to do if you get a positive test result.	9.0	0.0	≥3
A test that can be stored at room temperature.	9.0	0.0	0
Access to self-tests at a reasonable price for population groups with greater HIV risk.	8.8	0.4	≥3
Information that is accessible and understandable for everyone, including minors, people with low education levels or who are illiterate.	8.8	0.4	Young 7.5
A test that is easy to use.	8.6	0.5	≥3

The instructions on how to use the self-test and how to interpret test results need to be clear and comprehensible for all users, including those who do not have a high educational level.	8.6	0.9	≥3
The instructions must explain the implications of the window period preceding HIV seroconversion.	8.6	0.9	FWI 8.7
Propose an easy link without multiple intermediaries to get test results confirmed and to access to care if you get a positive result.	8.4	0.9	0
Situate self-tests as a complementary strategy with regard to existing screening options.	8.4	1.3	≥3
The instructions should include contact details on how to access community support or healthcare at a local level.	8.3	1.3	≥3
The instructions must be available in the foreign languages spoken in mainland France and in the French Overseas Departments (Guiana, Guadeloupe, Martinique, Reunion Island, Mayotte…).	8.2	1.1	FWI 8.3
Conduct specific campaigns promoting the self-test with the different high-risk population groups and taking into account their specificities.	8.2	1.3	≥3
Do not over « medicalize » the way the self-test is presented.	8.0	1.4	Trans 7.0
Public information concerning self-tests should be part of a general campaign promoting HIV screening and situating self-tests as one of a number of available test options, all of which have their specific advantages.	8.0	2.2	≥3
Self-tests should be widely accessible, not just in drugstores.	7.8	1.6	≥3
Accessible everywhere geographically.	7.8	1.6	SU 8.4
Information that it adapted and translated into the many languages spoken in Guiana.	7.8	1.8	0
Communicate about the self-test to professional and community organizations before communicating to the general public. Make healthcare stakeholders aware of the added value of self-testing with regard to individual prevention strategies, of its place in the screening system and access to care in the event of positive results.	7.7	1.3	≥3
Prepare and train those who will be selling or distributing self-tests (drugstores, community organizations…) or who provide support in using self-tests.	7.7	1.3	≥3
Work with cultural mediators on the different terms/symbols to employ.	7.6	1.3	0
Underline the advantages of self-testing: practical, rapid, discreet.	7.6	1.7	0
The instructions should include general information about HIV, HIV transmission and prevention.	7.4	1.7	≥3
A support service also available in foreign languages.	7.4	2.6	Trans 8.4
Access to care via the community organizations that provide HIV rapid testing.	7.4	3.0	0
Evaluate and, if need be, adapt how self-test users are received in drugstores.	7.3	1.9	0
Find ways of talking about HIV without being stigmatizing. Give users the words they will need to dare talk about it.	7.2	2.0	≥3
State people's rights and duties with regard to HIV self-testing.	7.0	1.2	FWI 8.0
When accessing self-tests, the users should be encouraged to get tested for other STIs, particularly in areas of high prevalence.	7.0	1.6	0
Launch the self-test with a national campaign on HIV screening for the general public.	7.0	1.9	0
Ensure the possibility of being physically accompanied by a community organization to access care.	7.0	3.1	0

[1]A zero (0) indicates a recommendation that is specific to this population. In cases in which the recommendation is common to only one other expert group, this group is indicated with its mean score. "≥3" means that this recommendation was brought up by two or more other groups of experts (amongst these recommendations brought up by ≥3 groups, those with an overall mean score ≥7 are also presented in Table S1).
SD: standard deviation; MSM: men who have se≥3 with men; MiSSA: migrants from sub-Saharan Africa; SU: substance users; Trans: transgender people; FWI: French West Indies; Guiana: French Guiana; Young: young people; Gen Pop: general population.

Table H: Shared and specific recommendations made by the ≥3 experts for young people and scoring ≥7

Recommendations	Mean	SD	Other groups that considered this reco.[1]
The instructions must indicate what to do if you get a positive test result.	9.0	0.0	≥3
Create a telephone hotline providing support for users who get positive test results, accessible 24/7.	9.0	0.1	SU 6.3
The instructions include information on how to access 24/7 free-of-charge telephone support on using the self-test, similar to that currently provided by Sida Info Service[2].	8.9	0.3	≥3
Evaluate self-test usage.	8.8	0.4	≥3
The instructions need to indicate how to read test results, including results that are unclear or invalid.	8.7	0.5	≥3
The instructions on how to use the self-test and how to interpret test results need to be clear and comprehensible for all users, including those who do not have a high educational level.	8.5	0.7	≥3
The instructions should indicate how to interpret a negative result and understand the implications of testing during the seroconversion window period.	8.5	1.0	≥3
Create a telephone hotline providing support on how to use the test, accessible 24/7.	8.5	1.2	≥3
The instructions need to be sufficiently attractive to be read even by those who think they already know how to use the self-test.	8.4	0.8	MSM 7.6
An online self-test assistance service (chat / SMS), 24/7.	8.4	0.8	≥3
Prepare and train those who will be selling or distributing self-tests (drugstores, community organizations…) or who provide support in using self-tests.	8.3	0.6	≥3
A support service that can direct the person who gets a positive test result to a clearly identified care and support network (especially for young people who do not have an easy or well established link with the care system).	8.3	0.8	≥3
The instructions should address the question of the need for support if a test result is positive: who should I speak to? Listing the different possible sources for accessing support.	8.3	0.8	≥3
The instructions must make it clear that you should only buy self-tests that are approved for use in France.	8.3	0.9	0
The instructions should use pictograms.	8.2	0.6	≥3
The instructions need to explain that saliva cannot transmit the virus, even if you can diagnose HIV using saliva.	8.2	1.0	≥3
Public information concerning self-tests should be part of a general campaign promoting HIV screening and situating self-tests as one of a number of available test options, all of which have their specific advantages.	8.2	1.0	≥3
The instructions must underline the importance of continuing to protect yourself, using condoms and sterile equipment.	8.1	1.1	≥3

The instructions should inform minors about their right to confidentiality in accessing health care.	8.1	1.6	0
Propose supervised self-testing; however this should be an option: supervision should not be a precondition for accessing the test.	8.0	1.2	0
Inform minors about their right to confidentiality when self-testing.	7.9	1.7	0
Situate self-tests as a complementary strategy with regard to existing screening options.	7.9	1.8	≥3
The instructions should underline the importance of knowing if you are HIV positive as early as possible. An optimistic approach to HIV care and living with HIV.	7.8	1.0	≥3
The instructions should be culturally adapted to the different communities affected by HIV and validated with these communities.	7.8	1.3	≥3
Underline the importance of only using self-tests that are approved for use in France.	7.8	2.3	≥3
Inform users about the reliability of self-tests (sensitivity and specificity).	7.7	0.5	≥3
Create community support networks for young people, whatever their test result might be (positive or negative).	7.7	1.0	FWI 8.8
The instructions need to mention of the possibility of getting a false positive result.	7.7	1.8	0
The instructions should include general information on HIV, HIV transmission and prevention.	7.6	1.6	≥3
Self-test outlets, including sales outlets (drugstores, etc.) must be able to provide oral information by personnel trained to inform and assist with self-test use.	7.5	1.2	≥3
Communicate with health professionals and community organizations ahead of communicating with the general public. Make health professionals aware of how the self-test will contribute to individuals' prevention strategies, its position with regard to other screening options and how best to access care if test results are positive.	7.5	1.8	≥3
The instructions need to emphasize that a negative test result does not mean that your partner is negative, even if you had unprotected sex.	7.5	1.8	≥3
Make the most of the self-test being new and easy to use to sensitize young people to HIV screening.	7.5	2.2	0
Information that is accessible and understandable for everyone, including minors, people with low education levels or who are illiterate.	7.5	2.4	Guiana 8.8
Zero cost for the user to access telephone support services, including from smartphones.	7.5	2.5	≥3
Provide support and information on the administrative aspects of accessing health and social care should you get a positive test result.	7.4	1.4	0
The instructions should inform users about their rights and responsibilities with regard to HIV self-testing.	7.4	1.7	≥3
The instructions should provide complementary information on STIs and viral hepatitis, and how to screen for them, emphasizing the fact that they cannot be detected by the HIV self-test.	7.4	1.8	≥3
Provide a variety of different ways of accessing self-tests (for young people), taking into account cultural and geographical diversity and in terms of cost.	7.4	2.3	≥3
The self-test should be easy to access, and at low cost or free-of-charge, for people under 25 who refuse other forms of screening.	7.4	2.5	0
A moderate and accessible price for all.	7.3	2.2	≥3
Official government texts need to make it clear that minors have the right to access self-tests.	7.2	2.2	MiSSA 6.1
Train all the principle stakeholders working with young people in the community on sexuality and STIs.	7.1	2.1	0

Self-tests should be available free-of-charge in school infirmaries.	7.1	2.3	0
The instructions should orientate users to an internet site where they can see videos of the test being used.	7.0	1.7	≥3
The instructions should contain information about all ways of screening for HIV and how to access them.	7.0	2.1	0
Encourage self-tests using cravicular samples ("saliva" tests – e.g. OraQuick®).	7.0	2.3	0

[1]A zero (0) indicates a recommendation that is specific to this population. In cases in which the recommendation is common to only one other expert group, this group is indicated with its mean score. "≥3" means that this recommendation was brought up by two or more other groups of experts (amongst these recommendations brought up by ≥3 groups, those with an overall mean score ≥7 are also presented in Table S1).

[2]Sida Info Service: AIDS Info Service, a government-funded hotline providing information and support on HIV/AIDS in France

SD: standard deviation; MSM: men who have se≥3 with men; MiSSA: migrants from sub-Saharan Africa; SU: substance users; Trans: transgender people; FWI: French West Indies; Guiana: French Guiana; Young: young people; Gen Pop: general population.

Table I: Shared and specific recommendations made by the experts for the general population and scoring ≥7

**Recommendations**	**Mean**	**SD**	**Other groups that considered this reco.**[1]
The instructions must indicate what to do if you get a positive test result.	8.9	0.3	≥3
The instructions on how to use the self-test and how to interpret test results need to be clear and comprehensible for all users, including those who do not have a high educational level.	8.6	0.7	≥3
The instructions need to indicate how to read test results, including results that are unclear or invalid.	8.5	0.7	≥3
The instructions should be sufficiently concise to be easily readable in one sitting.	8.3	1.3	≥3
Access to self-tests at a reasonable price or free-of-charge for population groups with greater HIV risk.	8.1	1.2	≥3
The instructions include information on how to access 24/7 free-of-charge telephone support on using the self-test, similar to that currently provided by Sida Info Service[2].	7.9	1.2	≥3
The instructions should address the question of the need for support if a test result is positive: who should I speak to? Listing the different possible sources for accessing support.	7.9	1.9	≥3
A test that is easy to use.	7.8	1.5	≥3
Evaluate linkage to care for people who discover positive test results using self-tests.	7.7	0.9	Trans 8.9
A moderate and accessible price for all.	7.7	1.6	≥3
Create a telephone hotline providing support on how to use the test, accessible 24/7.	7.7	2.5	≥3
Ensure regular self-test quality control.	7.6	1.4	0
Evaluate self-test usage.	7.5	0.7	≥3
The instructions should indicate how to interpret a negative result and understand the implications of testing during the seroconversion window period.	7.5	0.8	≥3
Prefer the most reliable tests (in terms of sensitivity and specificity).	7.5	2.4	MSM 8.9

Public information concerning self-tests should be part of a general campaign promoting HIV screening and situating self-tests as one of a number of available test options, all of which have their specific advantages.	7.2	1.8	≥3
The self-test needs to be available to community organizations via a central purchasing mechanism.	7.2	1.9	0
The instructions should use pictograms.	7.1	2.3	≥3
The instructions should emphasize the importance of privacy, and the need to think carefully before disclosing the test result to anyone.	7.0	1.2	0
The instructions must underline the importance of continuing to protect yourself, using condoms and sterile equipment.	7.0	1.4	≥3

[1]A zero (0) indicates a recommendation that is specific to this population. In cases in which the recommendation is common to only one other expert group, this group is indicated with its mean score. "≥3" means that this recommendation was brought up by two or more other groups of experts (amongst these recommendations brought up by ≥3 groups, those with an overall mean score ≥7 are also presented in Table S1).

[2]Sida Info Service: AIDS Info Service, a government-funded hotline providing information and support on HIV/AIDS in France

SD: standard deviation; MSM: men who have se≥3 with men; MiSSA: migrants from sub-Saharan Africa; SU: substance users; Trans: transgender people; FWI: French West Indies; Guiana: French Guiana; Young: young people; Gen Pop: general population.
